# 93096 Does gender matter? Gender differences in the relationship between resting-state functional connectivity and emotion regulation in alcohol use disorder.

**DOI:** 10.1017/cts.2021.507

**Published:** 2021-03-30

**Authors:** Kai Xuan Nyoi, Emily Koithan, Timothy Hendrickson, Hannah Verdoorn, Casey Gilmore, Bryon O. Mueller, Matt Kushner, Kelvin O. Lim, Jazmin Camchong

**Affiliations:** 1 University of Minnesota Department of Psychiatry and Behavioral Sciences; 2 University of Minnesota Informatics Institute; 3 Minneapolis VA Health Care System

## Abstract

ABSTRACT IMPACT: Our research has the potential to impact human health by identifying gender specific neural markers of emotion regulation in alcohol use disorder. OBJECTIVES/GOALS: Emotion dysregulation is known to be mediated by altered functional organization of the limbic system in addiction. This preliminary study sought to identify gender effects in the association between emotion regulation and resting-state functional connectivity (rsFC) of a negative affect network. METHODS/STUDY POPULATION: 55 individuals receiving treatment for alcohol use disorder (˜2 weeks of abstinence) were recruited for this study and included in this analysis (N=55; Age: M=41.78, SD=10.66; 21 females). RsFC within a network involved in the withdrawal/negative affect stage of addiction and Personality Inventory for DSM-5 (PID-5) metrics were collected from all participants. RsFC data were preprocessed using the Human Connectome Project pipelines. Correlations between (a) rsFC within the withdrawal/negative affect network and the (b) scores of the negative affect subscale of the PID-5 instrument were conducted for each gender separately. RESULTS/ANTICIPATED RESULTS: Independent samples t-test showed a statistically significant gender difference in the PID-5 negative affect scores (Males: M=1.02, SD=0.66; Females: M=1.53, SD=0.51); t(55)=-3.002, p=0.004. Only females showed a significant correlation between rsFC within the withdrawal/negative affect network and negative affect scores of the PID-5 (r=0.51, p<0.05). Fisher r-to-z test showed significant gender differences (z=-1.91; p=0.03, 1-tailed) in correlations coefficients representing the relationship between rsFC of the withdrawal/negative affect network and negative affect (PID-5 subscale). DISCUSSION/SIGNIFICANCE OF FINDINGS: Preliminary findings suggest that the relationship between neural networks mediating emotion regulation and negative affect is only found in females. These results provide valuable data to inform personalized chemical dependency treatment that targets emotion regulation specific to females.

